# Alternate actions of CDK4/6 inhibitors beyond cell cycle blockade: unexplored roles in therapy resistance 

**DOI:** 10.1007/s10555-025-10307-w

**Published:** 2025-12-12

**Authors:** Domenica Scordamaglia, Marianna Talia, Azzurra Zicarelli, Adelina Assunta Mondino, Salvatore De Rosis, Marika Di Dio, Francesca Silvestri, Chiara Meliti, Francesca Cirillo, Ernestina Marianna De Francesco, Roberta Malaguarnera, Carlo Capalbo, Marcello Maggiolini, Rosamaria Lappano

**Affiliations:** 1 Department of Pharmacy, Health and Nutritional Sciences, University of Calabria, Rende, 87036 Italy; 2 Department of Medicine and Surgery, University of Enna “Kore,”, Enna, 94100 Italy; 3 Medical Oncology Unit, Annunziata Hospital Cosenza, Cosenza, 87100 Italy

**Keywords:** CDK4/6 inhibitors, Resistance, Cellular senescence, Metabolic reprogramming, Tumour microenvironment, Immune surveillance, Cancer-associated fibroblasts

## Abstract

Cell cycle dysregulation and the aberrant activation of cyclin-dependent kinases (CDKs) lead to uncontrolled cell proliferation; therefore, these events represent well-established hallmarks of cancer. The advent of CDK4/6 inhibitors, namely, palbociclib, ribociclib and abemaciclib, has changed the management of oestrogen receptor (ER)-positive/HER2-negative advanced breast tumours. The clinical success of these drugs for the treatment of breast cancer has encouraged diverse clinical trials aimed at exploring novel combinatorial regimens of CDK4/6 inhibitors in different types of tumours. Hence, a comprehensive understanding of the mechanisms of action of these agents is essential to extend their benefits. Emerging evidence suggests that CDK4/6 inhibitors exert antitumour activity through other mechanisms beyond the acknowledged ability to block the cell cycle, including the induction of stress response pathways, the reprogramming of cancer cell metabolism, the modulation of the tumour microenvironment, the enhancement of the antitumour immune responses and the reduction of immune evasion. Nonetheless, the acquired resistance to CDK4/6 inhibitors remains a major therapeutic challenge. Thus, the identification of molecular drivers involved in the resistance to these drugs is crucial for the design of novel therapeutic approaches and the selection of patient-centred strategies in various types of tumours.

## Background

Fine-tuned cell division is fundamental for the development of normal tissues. As such, several checkpoints are involved in the progression of the cell cycle to prevent the transmission of genetic damage to new generations of cells [1]. The failure of these regulatory mechanisms leads to aberrant cell division, which is recognized as a hallmark of cancer [2].

The mitogen-driven cell cycle progression is orchestrated by transcriptional programs and protein degradation that modulate the timely accumulation of proteins, named cyclins, which in turn bind to and activate the cyclin-dependent kinases (CDKs), which are key regulators of cell cycle entry, progression and termination [3]. To date, the dysregulated expression and activity of either cyclins or CDKs are associated with the mitogen-independent cell proliferation and the bypass of checkpoints designed to prevent the effects of genomic damage, therefore prompting diverse types of cancers [4]. These observations paved the way for the development of CDK inhibitors as promising agents for cancer therapies [2]. The first generation of ATP-competitive pan-CDK inhibitors has been replaced by next-generation selective inhibitors of CDKs, especially CDK4 and CDK6, named palbociclib, ribociclib and abemaciclib [5, 6]. The improved safety profile of CDK4/6 inhibitors compared with first-generation molecules provides a rationale for their use in the treatment of different tumours, either as monotherapy or in combination with inhibitors of various signalling pathways [7]. In particular, palbociclib, ribociclib and abemaciclib have been approved by the Food and Drug Administration (FDA) and the European Medicines Agency (EMA) for the treatment of oestrogen receptor (ER)-positive and human epidermal growth factor receptor 2 (HER2)-negative advanced-stage or metastatic breast cancers in combination with endocrine therapy [2, 8, 9]. Worthy, preclinical studies on CDK4/6 inhibitors have provided promising results in malignancies other than breast cancer [10–12]. Likewise, several clinical studies are ongoing to investigate the potential usefulness of CDK4/6 inhibitors in the management of diverse malignancies either used alone or in combination with other therapeutic approaches (NCT03170206; NCT05358249; NCT02905318; NCT03891784; NCT03114527; NCT04391595; NCT05617885; NCT03675893; NCT02664935; NCT05935748; NCT03024489; NCT03709680; NCT03310879; NCT06307249; NCT06328049 and NCT05865132). In this context, a comprehensive understanding of the mechanisms underlying the antitumour effects of CDK4/6 inhibitors is crucial. The key event enabling cell cycle entry relies on the accumulation of cyclin D-CDK4/6 complexes, which are involved either in the regulation of the re-entry of cells into the cycle after a reversible arrest in quiescence caused by mild DNA damage, or in the permanent cell cycle arrest known as senescence occurring upon severe DNA damage [1]. Cyclin D-CDK4/6 complexes promote cell cycle entry by phosphorylating and thereby inactivating the retinoblastoma (Rb) tumour suppressor protein, a key inhibitor of the E2F transcription factor [12]. In both normal and cancer cells, the phosphorylation of Rb destabilizes its interaction with E2F, thereby activating an E2F-dependent transcriptional program towards the regulation of genes involved in cell cycle progression, DNA replication and cell growth [13]. By binding to and inhibiting CDK4/6 activity, palbociclib, ribociclib and abemaciclib prevent the phosphorylation of Rb, keeping it in an active growth-suppressive form. Therefore, E2F remains bound to Rb, and the cell cycle is arrested in G1 phase, hence preventing cell proliferation [14]. In addition to their well-established roles in promoting mitotic cell cycle entry through the regulation of Rb function, CDK4/6 have also been implicated in several non-cell cycle-related processes [16–19]. Here, we provide an updated overview of the additional mechanisms through which CDK4/6 inhibitors may exert antitumour effects on breast cancer and other types of cancers. For instance, these actions may include the induction of tumour cell senescence, the enhancement of antitumour immune surveillance and the metabolic reprogramming of the tumour microenvironment (TME). A better understanding of these mechanisms is crucial for developing innovative strategies aimed at optimizing the use of CDK4/6 inhibitors, even in tumours with Rb deficiency or mutation, which are usually resistant to these agents [19]. It should be also noted that many patients initially respond to CDK4/6 inhibitors but subsequently develop resistance, highlighting the need for further in-depth investigations of the underlying molecular mechanisms [20]. In this context and consistent with recent evidence showing that the tumour microenvironment may limit the efficacy of a wide variety of breast cancer therapeutic approaches, including CDK/46 inhibitors [22–27], we also discuss evidence regarding the capacity of CDK4/6 inhibitors to induce the release of protumourigenic senescence-associated molecules as well as to exert context-dependent stimulatory effects on important TME components, namely, cancer-associated fibroblasts (CAFs). Overall, the data discussed highlight the urgent need to circumvent the resistance to CDK4/6-targeted therapies towards the identification of effective and tailored combination treatments.

## CDK4/6 inhibitor-induced senescence

Cellular senescence is described as a stable and prolonged state of proliferative arrest triggered by damaging events, even in the presence of favourable growth conditions [27]. Of note, emerging evidence suggests that senescent cells can escape permanent growth arrest and resume proliferative activity, challenging the notion of senescence as an irreversible state [27]. Senescent cells exhibit peculiar morphological alterations, such as an increased cell size and lysosomal mass, cytoskeletal and membrane modifications, increased mitochondrial fusion and nuclear changes [27]. In this context, a short exposure of SK-MEL-103 metastatic melanoma cells to palbociclib resulted in stable proliferative arrest, which was attributed to drug sequestration within lysosomes [28]. Mechanistically, palbociclib trapping triggered a sustained autocrine and paracrine senescence, and these effects were enhanced using lysosome-targeting agents [28]. Recently, Nehme and colleagues reported that the increase in lysosomal mass induced by abemaciclib in breast cancer cells can increase sensitivity to lysosomotropic compounds, such as L-leucyl-L-leucine methyl ester and salinomycin [29]. In accordance with these findings, lysosomal-targeting drugs have been shown to resensitize abemaciclib-resistant breast cancer cells [30]. Although CDK4/6 inhibitors cause stable cell cycle arrest, previous experimental evidence has revealed that cells may re-enter the cell cycle in certain contexts, thereby contributing to the failure of treatments and the acquisition of a CDK4/6 inhibitor-resistant phenotype [32, 33]. In this context, different therapeutic regimens are currently under evaluation in both preclinical and clinical studies to ensure durable responses and prevent tumour relapse [34–43]. For instance, the use of abemaciclib in combination with doxorubicin downregulated the expression of a critical regulator of intracellular pH homeostasis, namely adenosine triphosphatase H^+^transporting accessory protein 2, leading to lysosomal dysfunction and senescence in breast cancer cells [43]. Considering the key role of mTOR as a regulator of lifespan, ageing and cellular senescence, diverse studies aimed to explore the efficacy of the simultaneous inhibition of CDK4/6 and mTOR [45–47]. For example, palbociclib in combination with mTOR inhibitors (i.e. rapamycin and everolimus) or the dual mTOR/phosphatidylinositol 3-kinase (PI3K) inhibitor PF04691502 enhanced senescence in both*in vitro* and *in vivo*non-small cell lung cancer models [47]. Similarly, the senescence triggered by palbociclib was amplified by rapamycin through an mTOR/mTORC1-dependent mechanism in melanoma cells [45]. Consistent with these findings, everolimus and the PI3Kα-specific inhibitor BYL719 have been reported to restore the sensitivity of resistant breast cancer cells to palbociclib [48]. In addition, combination treatments with palbociclib and the MEK inhibitor trametinib induced Rb-mediated senescence in KRAS-mutant lung cancer cells [33]. In MCF7 breast cancer cells, these agents led to an irreversible senescent state that persisted after drug withdrawal, in contrast to single-agent treatments, which induced a reversible senescent state [31]. Although the primary therapeutic challenge of CDK4/6 inhibitors relies on their limited success in treating triple-negative breast cancer (TNBC) [49], various combination strategies have proven efficacy in preclinical studies [36, 37, 51]. For example, the simultaneous administration of palbociclib with a dual inhibitor of STAT3 and CDK2, named nifuroxazide, induced senescence in MDA-MB-231 TNBC cells and reduced tumour growth in TNBC xenograft models [35].

Senescent cells exert a wide range of biological effects like tissue remodelling and repair, as well as inflammation and immune system modulation, through the release of cytokines, chemokines, growth factors and extracellular matrix (ECM) proteases [27]. These molecules, which are known as the senescence-associated secretory phenotype (SASP), can actively reshape the local microenvironment and modulate the immune surveillance [52, 53]. Recent findings have highlighted the beneficial effects of the SASP in hepatic tumour-initiating cells undergoing senescence in response to palbociclib treatment [53]. The resulting senescent cells secreted SASP-associated products that recruited and activated macrophages, thereby promoting antitumour immune responses [53].

Nevertheless, accumulating evidence suggests that persistent SASP-mediated signalling may also have detrimental effects on both normal and cancer cells [28, 55]. Remarkably, the production of SASP-related molecules triggered by CDK4/6 inhibitors enhances cancer cell proliferation, migration, invasion and immunosuppressive responses [22, 56–58]. For instance, transcriptomic analyses of non-small cell lung cancer cells treated with palbociclib revealed the upregulation of the SASP-related chemokine CCL5, which is involved in increasing the motility and invasiveness of malignant cells [57]. Gallanis et al. observed that palbociclib promotes lung metastasis in CDK4/6 inhibitor-resistant syngeneic breast cancer models, an event abrogated by the elimination of senescent cells [21]. Notably, these detrimental effects may arise not only from the cancer cell-intrinsic SASP but also from paracrine actions on cancer cells elicited by TME-derived SASP molecules. In addition, palbociclib may trigger in endothelial cells a senescent phenotype that contributes to enhanced tumour cell migration and immunosuppressive events [21]. Similarly, coculture assays of melanoma cells with CDK4/6 inhibitor-treated fibroblasts promoted the growth of neoplastic cells in diverse preclinical models and suppressed antitumour immune responses [56]. These observations suggest that the combination of CDK4/6 inhibitors and senolytic drugs might be an effective therapeutic strategy to counteract the onset of CDK4/6 inhibitor resistance. Accordingly, delayed tumour growth and reduced metastases were observed in MDA-MB-231 orthotopic TNBC model mice treated with palbociclib and the senolytic agent navitoclax [36]. Similar effects were reported in head and neck squamous cell carcinoma (HNSCC) cell lines, in which palbociclib-induced BCL-xL-dependent senescence caused a decrease in cell survival and an increase in apoptosis in the presence of navitoclax [37]. Taken together, these findings suggest that CDK4/6 inhibitor-induced senescence can either contribute to tumour suppression or promote therapy resistance and disease relapse, depending on the specific combination therapy employed and the cellular context. Hence, modulating the balance between induction and prevention of senescence remains crucial to achieve durable antitumour responses. Therefore, the use of CDK4/6 inhibitors together with senolytic agents should be carefully evaluated to enhance therapeutic efficacy and overcome resistance.

## Apoptotic responses to CDK4/6 inhibitor treatment

CDK4/6 inhibitors induce apoptosis either alone or in combination with diverse therapies in many cancer types, including lymphomas and breast, lung, bladder, pancreatic, prostate and renal tumours [59–63]. These effects involve molecular pathways that may be either dependent or independent of the classical CDK4/6-Rb signalling axis [64–68]. For example, transcriptomic analyses of oral squamous cell carcinoma revealed that the senescent phenotype induced by palbociclib may rely on both the CDK4/6/pRb/c-Myc/CDC25A signalling pathway and the downregulation of the DNA repair regulatory protein RAD51 toward the inhibition of DNA repair and, consequently, cellular senescence and apoptosis [68]. An additional mechanism related to apoptosis, independent of the classical CDK4/6-Rb signalling, has been described in lung squamous cell carcinoma, where palbociclib induced apoptosis by inhibiting the Src/STAT3 axis [63]. Further investigations have demonstrated that palbociclib can induce apoptosis both*in vitro* and *in vivo*[64]. In particular, the treatment of T24 bladder cancer cells with palbociclib resulted in hallmark features of apoptosis, including membrane blebbing, caspase-3 activation and mitochondrial release of the apoptosis-inducing factor (AIF). Mechanistically, these effects were mediated by the activation of CDK2, the mitochondrial translocation of p-Rad9 and its interaction with Bcl-xl, leading to the subsequent Bak activation [64]. Additionally, in pancreatic cancer cells, palbociclib increased the expression levels of important apoptosis proteins, including PUMA, Bak and Bax [65]. The expression of proapoptotic BCL-2 family proteins has also been assessed in castration-resistant and neuroendocrine prostate cancer cell models which were treated with palbociclib or abemaciclib in combination with the PARP inhibitor olaparib, leading to a greater growth inhibition and apoptosis respect to the use of a single agent [69]. Continuous abemaciclib treatment also inhibited cell proliferation and triggered senescence and apoptosis in breast cancer cells, regardless of the ER status [59]. Furthermore, ribociclib has been shown to induce apoptosis in breast cancer cells, with an enhanced efficacy exhibited in combination with everolimus, hence indicating a synergistic effect. Additional studies have revealed that the anticancer action of ribociclib administered alone, both*in vitro* and *in vivo*, on thyroid and human cervical cancers is attributable to the induction of apoptosis [61, 63]. Unlike senescence, apoptosis represents a more definitive therapeutic outcome by promoting the irreversible elimination of malignant cells. Although both events may occur concomitantly, the induction of apoptosis by CDK4/6 inhibitors remains the most desirable therapeutic endpoint. In this vein, exploring the combination strategies that employ CDK4/6 inhibitors with other agents capable of promoting programmed cell death in cancer cells may pave the way to overcome resistance and enhance therapeutic efficacy.

## Autophagy modulation by CDK4/6 inhibitors

Therapeutic interventions frequently induce autophagy, which is a highly conserved process leading to cytoprotection through a decrease in cellular damage [70]. In particular, autophagy plays a complex and context-dependent role in cancer: (i) suppression of tumour initiation by clearing damaged molecules and organelles; (ii) promotion of tumour cell survival when cells are exposed to therapy-induced stress or limited nutrient availability [71]. Studies of myeloma cells have shown that abemaciclib can induce dose-dependent morphological changes, including cytoplasmic vacuolization, which is indicative of autophagy [72]. Similar events were observed in HeLa cells, where abemaciclib-induced vacuoles resembled autolysosomes and appeared to be generated in a manner dependent on progranulin, a protein known for its role in autophagosome‒lysosome fusion [73]. These effects were inhibited by bafilomycin A1 and enhanced by rapamycin or Beclin-1 overexpression, supporting a mechanistic link between abemaciclib and autophagy modulation [73]. In contrast, studies on TNBC cells revealed that abemaciclib-induced cell death was not associated with autophagy-dependent mechanisms, as evidenced by the lack of LC3-I to LC3-II conversion and acidic vesicular organelles (AVOs) [74], thus suggesting that the autophagic response to abemaciclib is highly context dependent. Many studies have highlighted a role for autophagy in chemotherapy resistance, showing that the autophagic flux may be increased during the resistance process in diverse malignant contexts [75]. Notably, in small cell lung cancer (SCLC), this effect was reversed when chemotherapy was combined with CDK4/6 inhibitors, which suppressed chemoresistance by inducing cell death through the lysosomal dysfunction and autophagy [76]. Consistent with these findings, autophagy has been shown to modulate the onset of senescence and to cooperate with apoptotic pathways in certain tumour contexts, therefore triggering cell death mechanisms in the presence of apoptotic pathways impairment [77]. Autophagy has also been observed to occur concomitantly with apoptosis and to facilitate the apoptotic response upon CDK4/6 inhibition in certain cell contexts [79, 80]. For instance, palbociclib induced autophagy and apoptosis in hepatocellular carcinoma cells through the PP5/AMPK axis [78], whereas abemaciclib and the Cox-2 selective inhibitor celecoxib induced both autophagy and apoptosis, toward a reduced proliferation and invasion of colon cancer cells [79].

In addition to its tumour-suppressive role, autophagy may also exert cancer-promoting effects by clearing damaged cellular components and promoting the survival of tumour cells and the resistance to therapies [75]. Consistent with these observations, Vijayaraghavan and coworkers demonstrated that the senescence of breast cancer cells in response to palbociclib activates autophagy as a compensatory mechanism [80]. In accordance with these observations, CDK4/6 inhibitors have synergistic effects with autophagy inhibitors such as hydroxychloroquine, Lys05, CQ, bafilomycin A1 and spautin-1, leading to irreversible growth arrest in cancer cells [81, 82]. Building on this rationale, recent clinical trials in patients with advanced ER-positive/HER2-negative breast cancer (NCT03774472 and NCT05953350) reported that low- and high-doses of palbociclib in combination with hydroxychloroquine are well tolerated and exhibit encouraging signs of clinical efficacy [83, 84]. Based on this rationale, further investigations on the molecular determinants and signalling pathways governing autophagy in response to CDK4/6 inhibitors are mandatory.

## CDK4/6 inhibitors and metabolic reprogramming of cancer cells

Cancer is characterized by profound metabolic alterations that support malignant aggressiveness and tumour progression [84]. This metabolic reprogramming enables cancer cells to evade immune surveillance and proliferate under hypoxic or nutrient-deprived conditions [84]. A well-known example of these metabolic changes is the Warburg effect, whereby cancer cells utilize glycolysis over oxidative phosphorylation, even in the presence of oxygen [84]. Furthermore, tumours frequently exhibit an increased glutamine dependency, rewire lipid metabolism and modulate mitochondrial function. These metabolic shifts, which are often driven by oncogenes, tumour suppressors and signals originating from the TME, actively contribute to cancer progression and resistance to therapy [85]. Accumulating evidence indicates that CDK4/6 inhibitors may influence cancer cell proliferation and survival by either affecting key metabolic pathways or triggering metabolic reprogramming. One of the most relevant metabolic effects of CDK4/6 inhibition concerns the regulation of glucose metabolism. Zhang et al. reported that palbociclib induces the stabilization of the rate-limiting enzyme of gluconeogenesis fructose 1,6-diphosphatase 1 (FBP1) by repressing MAGED1 expression in pancreatic cancer cells, leading to decreased glucose metabolism [86]. While the initial CDK4/6 inhibition suppresses glucose metabolism, resistant cells often compensate by enhancing glycolytic flux. For example, ER/HER2-positive breast cancer cells resistant to palbociclib displayed an increased dependency on glucose catabolism, whereas the drug sensitivity was restored by targeting glycolysis [87]. Moreover, the antitumour efficacy of palbociclib was enhanced through the inhibition of glucose metabolism in ER-positive/HER2-negative sensitive cells, which are characterized by elevated aerobic glycolysis [87]. Although ER/HER2-positive and ER/HER2-negative cells differ in their extent and timing of glucose dependency, the aforementioned studies may suggest that tumour metabolic characteristics could be considered either biomarkers to monitor the therapeutic responses or targets to overcome therapeutic resistance.

At a broader level, CDK4/6 inhibition has also been shown to trigger a metabolic reprogramming orchestrated by the MYC transcription factor. In particular, an accumulation of MYC protein occurred upon palbociclib treatment in colorectal carcinoma cells, leading to enhanced glucose, glutamine and amino acid metabolism, activation of the mTOR pathway and reduction of HIF-1α-mediated responses to hypoxia [88]. These findings reveal additional metabolic weaknesses of cancer cells following CDK4/6 depletion that could be deepened in combinatorial regimens.

Mitochondrial reprogramming driven by anabolic biosynthesis, calcium signalling, redox regulation and other mechanisms is recognized as a hallmark of cancer cells [89]. These adaptive pathways are tightly regulated at mitochondria-endoplasmic reticulum contact sites (MERCs) [90]. Ziegler and colleagues showed that long-term exposure to abemaciclib or CDK4 depletion prevents MERC formation, thereby inhibiting mitochondrial apoptosis in TNBC cells that are often resistant to CDK4/6 inhibition. These findings identified CDK4 as a main coordinator of metabolic and survival responses in TNBC cells [90], further suggesting that the metabolic vulnerabilities acquired by TNBC cells upon CDK4 inhibition could be exploited as potential therapeutic targets in order to overcome resistance and impair tumour survival.

Additional evidence regarding the involvement of CDK4/6 inhibitors in mitochondrial metabolism has emerged in BRAF^V600^melanoma cells, where palbociclib triggered a peculiar form of metabolic reprogramming [91]. This metabolic shift differs from the changes triggered by BRAF and MEK inhibitors, which were used to suppress tumour-promoting signals [92]. Specifically, CDK4/6 inhibition promoted a shift toward a more oxidative mitochondrial metabolism, which is characterized by increased reliance on glutamine and fatty acid oxidation as primary energy sources rather than glucose consumption [91]. This transition in the bioenergetic profile seems to be, at least in part, dependent on p53 activity and reflects a CDK4/6-specific mechanism driving tumour cell metabolism independent of the MAPK pathway inhibition [91].

Consistent with the established involvement of cell cycle-related proteins in glucose metabolism, a further action of CDK4/6 inhibitors involves their impact on glucose homeostasis [93]. A preclinical study demonstrated that palbociclib induces marked alterations in glucose metabolism in mouse models in an age-dependent manner [94]. In particular, significant glucose intolerance, along with reduced insulin secretion and morphological alterations in pancreatic β-cells, such as cytoplasmic vacuolization and signs of endoplasmic reticulum stress, were observed in young rats treated with high doses of palbociclib. These changes were not detected in adult rats, suggesting that the metabolic effects of CDK4/6 inhibition may be influenced by the proliferative state of β-cells [94]. Although CDK4/6 inhibitors are generally well tolerated from a metabolic perspective in adults, the abovementioned preclinical findings suggest potential age-related differences in glucose metabolism that warrant further investigation [94]. Further evidence of the involvement of CDK4/6 inhibitors in glucose metabolism comes from studies of pancreatic ductal adenocarcinoma, where pharmacological CDK4/6 blockade leads to a marked increase in both glycolytic activity and oxidative phosphorylation together with mitochondrial expansion and the increased production of ATP and reactive oxygen species [17]. These metabolic changes are assumed to be linked to the activation of mTORC1 signalling. Accordingly, the simultaneous inhibition of mTOR and CDK4/6 was shown to trigger an energy crisis leading to apoptosis [17]. These observations suggest that targeting simultaneously the CDK4/6 and mTOR pathways may enhance therapeutic efficacy, driving tumour cells into metabolic collapse [17].

Among the strategies used to increase the efficacy of CDK4/6 inhibitors, recent preclinical studies identified cyclin-dependent kinase 7 (CDK7) as a promising target to overcome the intrinsic resistance of TNBC to this class of agents [95]. Remarkably, co-targeting CDK4/6 and CDK7 reduced cholesterol biosynthesis by attenuating the activity of the transcription factor forkhead box M1 (FOXM1), ultimately impairing TNBC cell proliferation both*in vitro* and *in vivo*[95].

Taken together, these findings underscore the critical role of CDK4/6 in regulating cancer cell metabolism, highlighting how their inhibition may unveil metabolic vulnerabilities that can be therapeutically exploited to overcome resistance and improve patient outcomes.

## CDK4/6 inhibitors and immune cells

Accumulating evidence indicates that the TME influences cancer cell growth, the invasive and metastatic potential, as well as the response to pharmacological treatments [97, 98]. Several cell populations of both innate and adaptive immune systems infiltrate the TME. Depending on their functional phenotype, these cells can either support or suppress tumour growth in accordance with the dynamic process of cancer immunoediting, which encompasses the following three distinct phases: elimination, equilibrium and escape [98]. Although immunotherapy enabled the achievement of remarkable clinical benefits [100–105], certain subsets of patients fail to experience beneficial effects [105], providing the rationale for more in-depth studies aimed at optimizing the response to drugs targeting the immune system [106]. Emerging data indicate that CDKs may also be involved in the regulation of innate immunity responses [107]. In this vein, the profiling of 13,942 colon cancers revealed a differential expression of immune-related genes and TME cell infiltration depending on the CDK4/6 expression, hence contributing to the identification of markers useful to assess the response to immune checkpoint inhibitors (ICIs) [108].

In addition, previous data have indicated that CDK4/6 inhibitors can influence cancer-associated immune responses within the TME by interacting with both tumour and immune cells. Worthy, CDK4/6 inhibitors have shown the ability to orchestrate anti-tumour immunity and long-term anti-tumour effects, leading to immunogenic cell death (ICD) [110–114]. Of note, ICD is an acknowledged key mechanism of boosting immune response against cancer cells [114]. The effectiveness of ICD in promoting immune surveillance depends on the efficient presentation of tumour-associated antigens and neoantigens to CD8 + T cells by antigen-presenting cells (APCs), such as dendritic cells (DCs), B cells and macrophages [116, 117]. In this context, CDK4/6 inhibitors exhibited the capability to promote the maturation of DCs, thereby enhancing T cell priming and boosting antitumour immune responses in*in vitro* and *in vivo*models [118–122]. Conversely, the depletion of macrophages and DCs was observed in melanoma patients as a side effect of the continuous administration of a therapeutic regimen including CDK4/6 inhibitors [122]. In line with these results, Kumar et al. have recently demonstrated that DCs proliferation is reduced in the peripheral blood samples collected from eight metastatic breast cancer patients receiving palbociclib and endocrine therapy. Interestingly, the adoptive transfer of ex vivo differentiated bone-marrow DCs restored the antitumoral effect of palbociclib in combination with ICIs, thus supporting DC-based approaches to improve the efficacy of therapeutic regimens combining CDK4/6 inhibitors to immunotherapy [118].

In addition to their effects on DCs, CDK4/6 inhibitors modulate TAMs that are shifted from an M2-like phenotype typically associated with immunosuppression, toward an M1-like phenotype that conversely contributes to anti-tumour immunity [124–128]. In BRCA1-deficient murine breast cancer models, this phenotypic reprogramming was mediated by the activation of the stimulator of interferon genes (STING) pathway, which is an established driver of enhanced anti-tumour immune responses upon CDK4/6 inhibition [129–131]. Next, a recent trial has shown a correlation between macrophage abundance and cyclin E levels in metastatic ER-positive/HER2-negative breast cancer patients, suggesting that the immune TME may be useful in predicting CDK4/6 inhibitor efficacy [130].

Macrophages and other APCs contribute to the effective activation of T cells not only through antigen presentation but also through the expression of costimulatory molecules that are essential for the coactivation of T cell recognition and function [229]. For instance, the expression of the coactivators CD40, CD80 and CD86, as well as the major histocompatibility complex (MHC) class I and II molecules, was found to be upregulated in mature DCs and macrophages by CDK4/6 inhibitors in different cancer models [110, 124]. One of the most relevant immune-related effects of CDK4/6 inhibitors is their ability to promote effector T cell infiltration and to enhance their tumour-killing capacity. In this regard, several studies of tumour cells treated with CDK4/6 inhibitors have demonstrated increased expression of cytokines such as CCL5, CXCL9, CXCL10 and IL-15 which are involved in the recruitment of CD8 + T cells [56, 126, 128, 134–136]. Moreover, CDK4/6 inhibition up-regulated interferon-γ (IFN-γ) expression, which in turn enhanced effector T cell functions along with interleukin-2 (IL-2) by activating the nuclear factor of activated T cell (NFAT) [134]. In line with these findings, CDK4/6 inhibition has also been associated with either an increased expression of markers of T cell activation or reduced levels of exhaustion markers [122, 137, 138]. A recent preclinical study has also indicated the functional reprogramming of TAMs as a new mechanism through which abemaciclib may enhance intratumoral CD8 + T cell infiltration in breast cancers [228]. These findings were recently corroborated by a retrospective study enrolling patients with ER-positive/HER2-negative metastatic breast tumours. In these patients, CDK4/6 inhibitors triggered a robust immune response in terms of elevated levels of both CD4 + T cells and anti-tumour CD137 +/CD8 + T cells [135].

CDK4/6 inhibitors are also capable of promoting the development of a T cell memory phenotype, thereby enhancing long-term antitumour activity. In this regard, several studies have shown that short-term treatment with CDK4/6 inhibitors is sufficient to drive CD8 + T cells towards a memory phenotype [139, 140]. Interestingly, an increased number of memory CD8 + T cells is associated with improved responses to ICIs, suggesting that CDK4/6 inhibitors may serve as valuable agents in combined immunotherapy regimens [138]. Other evidence has indicated that CDK4/6 inhibitors can reduce the population of immunosuppressive regulatory T cells (Tregs), which contribute to tumour progression by promoting immunological tolerance [138, 142]. Notably, due to their higher expression levels of CDK6, Tregs are more susceptible to CDK4/6 inhibition respect to CD8 + T cells [140]. Interleukin-17A-secreting γδ T cells have recently been identified as mediators of resistance to the CDK4/6 inhibitors by modulating TAMs in ER-positive/HER2-negative breast cancer patients [141]. This expands the spectrum of T-cell subsets that may need to be targeted to overcome the resistance to CDK4/6 inhibitors.

In addition to their effects on T cells, recent findings have indicated that CDK4/6 inhibitors enhance the activation, infiltration and interaction of B cells with the CD4 +/CD8 + T cells [123]. In this context, the frequent overexpression of CDK4/6 and their association with poor prognosis in diffuse large B-cell lymphoma (DLBCL) and mantle cell lymphoma (MCL) warrant further investigations on CDK4/6 inhibitors as potential therapeutic options for these patients (NCT01739309, NCT02414724) [142]. Recently, Hu et al. demonstrated that PI3K inhibition enhances the anti-tumoural effect of palbociclib in cell-derived xenograft (CDX) and patient-derived xenograft (PDX) mouse models of lymphomas, further supporting future research for the assessment of combination therapies including CDK4/6 inhibitors [142]. In parallel, CDK4/6 inhibition hampers tumour progression, therefore promoting the accumulation and maturation of NK cells, a further critical component of the tumour immunosurveillance from which may arise the natural killer/T-cell lymphoma (NKTL) [34, 111, 146]. In this context, CDK4/6 inhibitors were able to restore the sensitivity to the XPO1 inhibitor selinexor through the suppression of CDK4/6-pRb-E2F-c-Myc pathway in an NKTL xenograft model [144]. The recruitment and activation of NK cells induced by CDK4/6 inhibitors may involve the SASP-like chemokines and cytokines, which are modulated by these drugs, as mentioned previously [34, 148].

In addition to the immune surveillance-promoting effects of CDK4/6 inhibitors on immune cells within the TME, these agents are also associated with immunological changes in tumour cells. Goel and colleagues reported that abemaciclib enhances antitumour immunity by upregulating the expression of MHC class I molecules in breast cancer cells [145]. These findings were further corroborated by subsequent studies showing that abemaciclib also increases the levels of MHC class II molecules in*in vivo*models of breast and colon cancer [121]. Other studies revealed that palbociclib can induce the expression of both MHC molecules and their associated ligands in breast cancer cells [146]. Comparable evidence has been obtained from diverse types of cancer cells, including melanoma, malignant pleural mesothelioma, Ewing sarcoma and cancers related to Kaposi sarcoma-associated herpesvirus (KSHV), Epstein–Barr virus (EBV) and gamma herpes viruses [150–153]. Mechanistically, the up-regulation of MHC molecules on the cancer cell surface upon treatment with CDK4/6 inhibitors was correlated with the immune surveillance-promoting effects of the interferon signalling [137, 148, 153, 154]. Investigations in ovarian cancer models have also revealed the involvement of the STING pathway in the up-regulation of IFN-γ-stimulated genes upon palbociclib treatment [152]. Consistently, a weakened antitumour response was observed in mice receiving STING-deficient fibrosarcoma cells [129]. Further corroborating these data, Wang et al. showed that palbociclib in combination with talazoparib increased CD8 T cells and natural killer (NK) in colorectal cancer models via the cGAS/STING signalling. Worthy, anti-PD‐L1 therapy enhanced the antitumoral effect of palbociclib and talazoparib in immunocompetent mice, further supporting the exploration of novel combinatorial strategies for colorectal cancer patients [38].

As previously observed in immune cells, CDK4/6 inhibitors may induce the upregulation of several costimulatory surface molecules in cancer cells [150]. Consistent with these observations, the increased expression of the costimulatory molecule CD83 was detected in patients with ER-positive metastatic breast cancer treated with palbociclib [119](NCT02778685). Additional mechanisms contributing to the increased immunogenicity of cancer cells treated with CDK4/6 inhibitors include the Rb-dependent activation of long terminal repeat (LTR) enhancers, which regulate genes involved in immune surveillance and antigen presentation [153]. Moreover, recent studies suggest that CDK4/6 inhibition can reverse the immune-refractory phenotype of certain tumour cells. In this context, palbociclib restored the immune responsiveness in tumour cells by downregulating the expression of NANOG, a marker of multiple aggressive and immune-resistant phenotypes [154]. In accordance with these data, CDK4/6 inhibitors were able to reverse a tumour immunosuppressive status in ER-positive/HER2-negative metastatic breast cancer patients [135].

Multiple preclinical studies have suggested that the immunomodulatory effects of CDK4/6 inhibitors may enhance the efficacy of ICIs, thereby providing a rationale for combination therapies in cancer patients [124, 148]. In a murine colon cancer model, CDK4 knockout along with antibodies against programmed death-ligand 1 (PD-L1) enhanced antitumour immune responses, whereas in melanoma model mice, CDK4/6 inhibition reversed the resistance to ICIs [158–160]. At the mechanistic level, CDK4/6 inhibitors up-regulated in tumour cells the expression of PD-L1, which is an established biomarker for ICI responsiveness in certain tumours [160–165]. In particular, the decreased proteasomal degradation of PD-L1 together with the activation of a Rb/NF-κB-mediated transcriptional network have been identified as molecular events mediating the up-regulation of PD-L1 in cancer cells upon treatment with CDK4/6 inhibitors [166–168]. Data collected from several clinical studies testing the effectiveness of CDK4/6 inhibitors used in combination with ICIs are quite controversial, especially for breast cancer. In particular, early clinical trials failed to show substantial benefits from this therapeutic combination in patients with metastatic ER-positive breast tumours [169, 170]. On the other hand, the NEWFLAME and PACE trials reported the activity of two regimens combining ICIs, CDK4/6 inhibitors and endocrine therapies [171, 172]. Interestingly, a single-case study highlighted the potential benefits of combining CDK4/6 inhibitors with ICIs. In this study, a patient with SMARCA4-deficient small-cell ovarian carcinoma who experienced failure of multiple treatments achieved a clinical response to the combination of abemaciclib and nivolumab, an ICI that targets the PD-1 receptor [170]. Based on these findings supporting the clinical importance of combination regimens, including CDK4/6 inhibitors and ICIs, ongoing clinical efforts aim to evaluate this therapeutic strategy in various tumours towards the identification of predictive biomarkers and patient selection. In this context, the phase 2 ORACLE-RIPA clinical study, focusing on the time-dependent immune modulation effects of CDK4/6 inhibitors in ER-positive/HER2-negative early breast cancer patients, would help toward the identification of immune-related signatures associated with better response to these drugs (NCT05766410).

## CDK4/6 inhibitors and adipocytes

Adipocytes are important components and active regulators of the TME due to their ability to secrete metabolic substrates, growth factors, adipokines and cytokines [171], which contribute to crucial cancer-related processes such as cell proliferation, invasion, neoangiogenesis, immune surveillance evasion and therapy resistance [171]. Once adipocytes and cancer cells interact, a bidirectional signalling network and a state of metabolic symbiosis occur, particularly under stress conditions such as hypoxia and energy deprivation. This crosstalk fosters a TME that supports tumour progression [172]. Accordingly, obesity has been widely recognized as an independent risk factor for cancer development and associated with increased tumour-related mortality [173].

Emerging evidence has highlighted the role of CDK4 and CDK6 in the regulation of adipocyte functions, such as insulin signalling, adipogenesis, thermogenesis and lipid metabolism [177–179]. Likewise, CDK4 and CDK6 have been implicated in metabolic disorders, including diabetes and obesity [177]. In particular, CDK4 has been shown to influence both adipocyte differentiation and function by activating peroxisome proliferator-activated receptor γ (PPAR-γ). Consistently, CDK4 inhibition reduced the adipogenic potential of primary mouse embryonic fibroblasts, indicating that CDK4 can promote adipogenesis and adipocyte function by interacting with PPAR-γ [175]. In addition, CDK6 inhibited the white-to-beige fat transition by suppressing the transcription marker RUNX1, making it a promising therapeutic target for obesity and related metabolic disorders [178]. Specifically, mouse models lacking CDK6 present improved energy expenditure, glucose tolerance and insulin sensitivity, as well as enhanced resistance to high-fat diet-induced obesity [179]. Consistent with these observations and previous findings revealing that*de novo* lipogenesis in WAT is suppressed in obese individuals, CDK6 was able to negatively regulate *de novo*lipogenesis in adipose tissue [183, 184]. Notably, a marked increase in lipogenesis and lipid accumulation was observed in the WAT of mouse models with impaired CDK6 activity, further corroborating the potential of CDK4/6 inhibition as a therapeutic strategy for obesity-related metabolic disorders [181]. Consistent with these observations, CDK4/6 inhibitors may play a role in diet-induced obesity, thus impacting the management of cancer patients [182]. For example, abemaciclib reduced fat mass without affecting lean mass in obese mice fed a high-fat diet. Mechanistically, abemaciclib prevented diet-induced obesity by blocking Rb phosphorylation in the neurons of the mediobasal hypothalamus, then promoting lipid oxidation [182]. Cancer-associated adipocytes (CAAs) have emerged as key components of the TME as they actively contribute to cancer progression through metabolic and paracrine interactions with tumour cells [171]. In this context, it should be mentioned that palbociclib counteracted the proliferative effects induced by the secretome of cancer-associated adipose tissue (CAAT) on breast cancer cells [183]. Specifically, proteomic analyses have revealed several CAAT-derived factors, such as leptin and insulin-like growth factor binding protein 2 (IGFBP2), which activate pro-oncogenic pathways, including the PI3K/AKT and STAT3, thereby promoting tumour growth and resistance to therapy [183]. Nevertheless, the molecular mechanisms by which palbociclib counteracts CAAT-induced cancer cell proliferation have not yet been elucidated.

Numerous clinical studies have assessed that obese patients, especially those diagnosed with breast cancer, likely develop resistance to endocrine therapies and chemotherapies compared to lean subjects [187–192]. Of note, the American Society of Clinical Oncology (ASCO) recently released an update of their previously published guidelines for the correct management of obese cancer patients, thus suggesting that more customized therapies for this category of patients are needed [190]. Undoubtedly, considering the patient-associated demographic characteristics such as obesity may help to unveil good-responder categories for specific treatments. For instance, overweight cancer patients are reported to obtain more benefits from ICIs administration compared to those with a lower body mass index (BMI) [191]. As a state of chronic low-grade systemic inflammation, obesity is associated with a pro-inflammatory immune environment that is hampered by CDK4/6 inhibitors [195, 196]. In line with this assumption, a recently published meta-analysis revealed that metastatic breast cancer patients with high BMI may benefit from the treatment with CDK4/6 inhibitors in terms of progression-free survival (PFS) [197, 198]. Considering that better PFS improvements were evidenced when obesity status was measured as visceral adipose tissue instead of BMI, the identification of universal biomarkers for adiposity in metastatic breast cancer is needed [197, 199]. Cumulatively, these findings underscore the potential of CDK4/6 inhibitors to disrupt paracrine interactions within the adipose-rich TME, highlighting new avenues for therapeutic intervention. Given that obesity is a well-established risk factor for breast cancer, even involving the activation of molecular signalling pathways similar to those driving tumour growth, targeting CDK4/6 may provide a unified strategy to counteract both obesity-associated metabolic alterations and cancer progression.

## CDK4/6 inhibitors and cancer-associated fibroblasts (CAFs)

Significant efforts have recently focused on the development of novel anticancer therapies targeting CAFs, which are the most abundant stromal cell type and a key component of the TME [200, 201]. This cell population, which is characterized by molecular and functional heterogeneity, is engaged in multifaceted communications among cancer cells and other TME components [202, 203]. Due to their ability to secrete cytokines, chemokines, growth factors and ECM components, CAFs actively contribute to the ECM remodelling as well as the metabolic and immune reprogramming of the TME toward tumour growth, invasiveness and metastasis [200]. In this respect, several findings support the rationale of combining CAF-targeting strategies with the CDK4/6 inhibition, especially for ERα-positive breast cancer. Accordingly, paracrine signalling from CAFs has been shown to reduce the expression and activity of ERα in luminal breast tumours and aggressive phenotypes [201]. In a high-throughput screen aimed at identifying compounds capable of counteracting these CAF-induced effects, abemaciclib and palbociclib emerged as potential agents able to restore ERα signalling in MCF7 breast cancer cells [22]. These findings are particularly important since the loss of ERα expression has been implicated in the resistance to CDK4/6 inhibitors, which is still a major clinical challenge in the treatment of ER-positive breast cancer [202]. In this context, the G protein-coupled estrogen receptor (GPER), which mediates estrogenic signalling in both CAFs and breast tumour cells, has been associated with the resistance of breast cancer cells to palbociclib [24, 207, 208]. In particular, elevated expression levels of GPER found in palbociclib-resistant cells have been shown to contribute to the proliferative features of these cells [23]. In addition, palbociclib activated the main molecular sensors of GPER signalling and to increase the expression of proinflammatory genes in CAFs in a GPER-dependent manner [23]. These findings along with data indicating the association of these proinflammatory genes with poor clinical outcomes of ER-positive breast cancer patients are consistent with the observation that palbociclib elicits reduced efficacy in breast cancer cells cocultured with CAFs [23]. Overall, these findings suggest that GPER-mediated signalling within the TME may contribute to the reduced effectiveness of CDK4/6 inhibitors, underscoring the need to consider stromal-tumour interactions to overcome therapeutic resistance. Further supporting the observations that certain transduction events in CAFs may contribute to palbociclib resistance in breast cancer, single-cell RNA sequencing analysis of biopsies collected from a patient before and after palbociclib treatment revealed an increased abundance of CAFs in metastatic samples [24]. In addition, a pivotal role of a distinct subpopulation of CAFs has been suggested in the resistance to CDK4/6 inhibitors. Specifically, CD63⁺ CAFs were found to be markedly enriched in ER-positive breast cancers resistant to the CDK4/6 inhibition. Mechanistically, this CAF subset was shown to compromise the efficacy of CDK4/6 inhibitors in both breast cancer cell lines and xenograft models through the exosomal delivery of miR-20, which in turn suppressed Rb expression [25]. Together, these findings emphasize that specific CAF subsets, such as CD63⁺ CAFs, play a role in shaping the therapeutic outcomes by driving the resistance to the CDK4/6 inhibitors through paracrine and exosome-mediated mechanisms.

Despite the frequent loss of Rb has limited the investigations on CDK4/6 inhibitors in TNBC, preclinical studies have shown that some TNBC models remain responsive to these agents [50, 209]. These studies provide a rationale for clinical trials including patients affected by this malignancy in order to explore the efficacy of the CDK4/6 inhibition alone and in combination with other chemotherapeutics or targeted therapies. To date, a major challenge to the use of CDK4/6 inhibitors in the treatment of TNBC remains the identification of both the molecular mechanisms underlying drug resistance and reliable biomarkers to stratify subsets of patients who could benefit from these agents. Consistent with several reports that the TME can drive therapeutic resistance and disease progression even in TNBC, tumour–stroma interactions have been shown to modulate the sensitivity of TNBC cells to diverse drugs, including CDK4/6 inhibitors [27, 210]. In particular, the ability of palbociclib to reduce the viability of basal-like TNBC cells was significantly blunted when the cells were cocultured with fibroblasts [26], suggesting that stromal fibroblasts within the TNBC microenvironment can impair the sensitivity to CDK4/6 inhibition. Altogether, these findings highlight that a deeper understanding of the crosstalk between CAFs and tumour cells is crucial to identify key mediators of resistance to CDK4/6 inhibitors toward the development of novel therapeutic strategies.

## Conclusions and perspectives

**Fig. 1:**
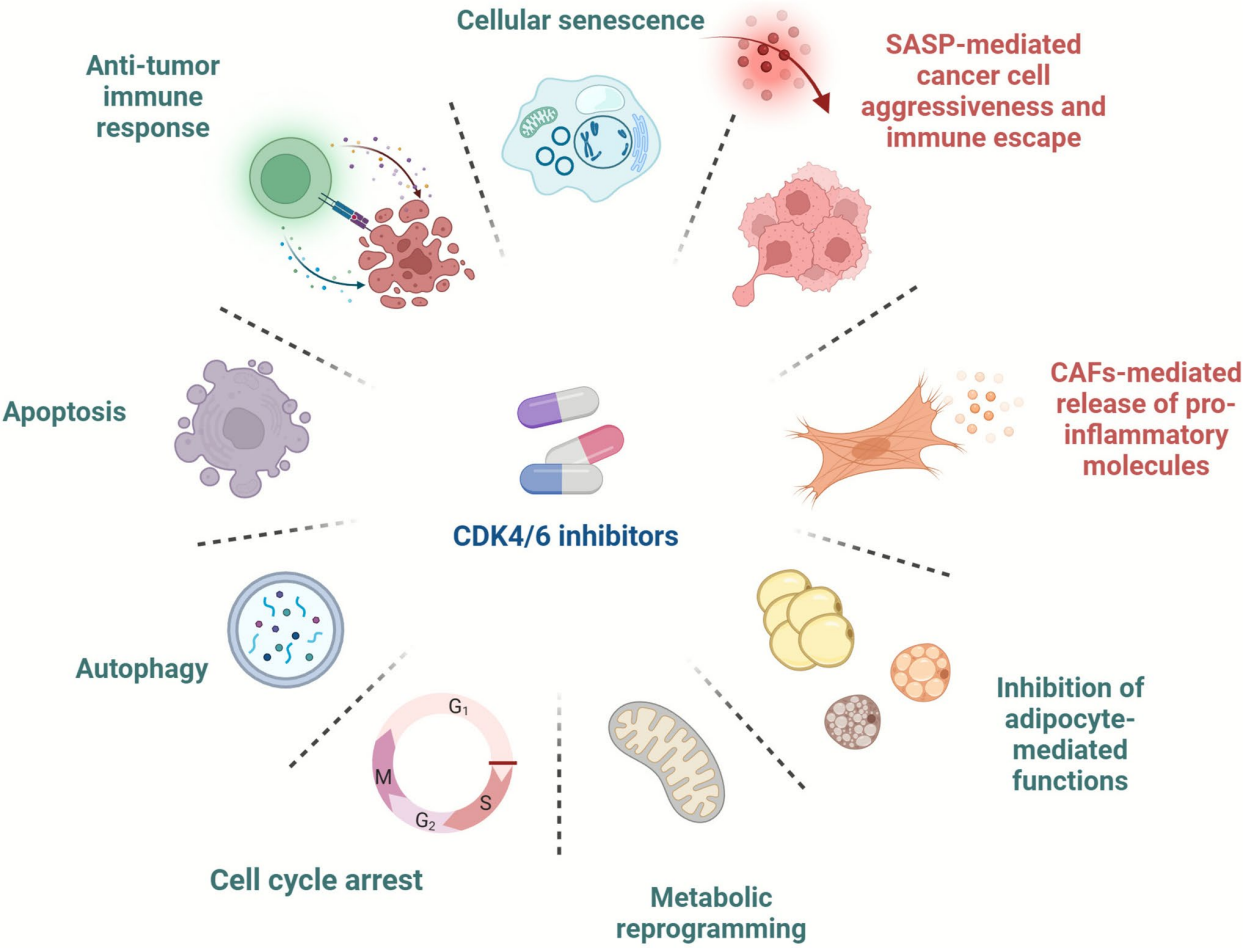
An overview of the cellular and molecular mechanisms underlying the alternate actions of CDK4/6 inhibitors beyond cell cycle arrest is presented, with explanatory details available in the text. The tumour-promoting mechanisms are shown in red, and the antitumorigenic mechanisms are shown in green. Created at https://BioRender.com

In addition to their ability to block the cell cycle by inhibiting Rb phosphorylation, CDK4/6 inhibitors are currently under deep investigation for their multifaceted anti-tumour activities beyond cell cycle regulation (Fig. 1; Table 1). In this vein, increasing evidence highlights the involvement of the CDK4/6 inhibition in cellular senescence, apoptosis, autophagy, cancer cell metabolism, antitumour immune surveillance and in reshaping the TME, opening new avenues for novel therapeutic strategies.

**Table 1:** Noncanonical biological activities of CDK4/6 inhibitors beyond cell cycle arrest and the underlying molecular mechanisms

	Abemaciclib	Palbociclib	Ribociclib
**Senescence**	Reduction of lysosomal biogenesis and decreased sensitivity to lysosomotropic agents in breast cancer cells [29] ANGPTL4-mediated geroconversion in liposarcoma cell lines [40] Reduced ATP6AP2 disrupts pH regulation and lysosomal function in breast cancer cells [43]	TGF-β-dependent activation of p21CIP1/p15INK4B in endothelial cells [21] Loss of phospho-RB1 and FOXM1 in melanoma cells [28] MYC degradation in breast cancer cells [31] Down-regulation of CCNE2, CCNA2, CCNB1, CCNB2, and up-regulation of CCND1, CCND2, CCND3 in TNBC cells [32] Increase of DEC1, DCR2 and CDKN2B levels in combination with trametinib in KRAS-mutant lung cancer cells [33] Ablation of MYC protein levels in combination with ERK MAPK inhibitor in pancreatic ductal adenocarcinoma [42] Inhibition of mTOR signaling in melanoma cells [45] mTORC activity in ER-positive breast cancer cells [46] Loss of Mdm2 expression in normal fibroblasts [56]	ATR or Chk1 dephosphorylation-dependent DNA damage response in ovarian cancer cells in combination with cisplatin [41]
**Apoptosis**	Suppression of CDK4/6-Cyclin D complex, ROS generation and depolarization of mitochondrial membrane potential in prostate cancer cells [66]	5′ AMPK activation and PP5 inhibition in hepatocellular carcinoma [78] Rb-independent STAT3 phosphorylation in lung squamous cell carcinoma cells [63] CDK2-induced Rad9-mediated reorganization of the Bak-Bcl-xl complex in bladder cancer cells [64] AMPKα and miR-33a upregulation inhibits fatty acid synthesis in pancreatic cancer cells [65] DNA damage and inhibition of DNA repair in oral squamous cell carcinoma [68]	Down-regulation of FOXM1, Cyclin A1 and Myc in anaplastic thyroid cancer cells [60] Down-regulation of CDK4, CDK6, E2F1, p-Rb and increased Bax expression in cervical cancer cells. Down-regulation of CDK4, CDK6, cyclin D1, Rb and Ki-67 in C33A xenografts [62]
**Autophagy**	Autophagosome-lysosome fusion in a PGRN-dependent manner in endometrial cancer cells [73] Up-regulation of ATG5, p62, LC3 and Beclin-1 in colorectal cancer cells [79] ROS/ERK axis in head and neck squamous cell carcinoma cells [81]	5′ AMP-activated protein kinase (AMPK) activation and protein phosphatase 5 (PP5) inhibition in hepatocellular carcinoma [78] AMBRA1 up-regulation induces CDK6 autophagic degradation, preventing TFEB/TFE3 activation and reducing lysosomal gene expression in small cell lung cancer [76] ROS production in breast cancer cells [80]	AMBRA1 up-regulation induces CDK6 autophagic degradation, preventing TFEB/TFE3 activation and reducing lysosomal gene expression in small cell lung cancer [76]
**Immune cells**	DC maturation via CSF2-driven M1 polarization [120] CD8⁺ T cell recruitment and activation via HIF-1α-mediated MIF increase [228] CXCL10 and CXCL13-dependent B cell activation, CD8⁺ T cell recruitment and activation and macrophage polarization and infiltration [123] CD8⁺ T cell recruitment and activation via IL-15-secreted Sell(hi) neutrophils [133] Macrophage polarization and infiltration through the MIF-CD44/CD74 axis [228] Macrophage polarization and infiltration via the CCL2-dependent recruitment of IL-17A-secreting γδ T cells [141] Mxd4/Myc-dependent induction of a T cell memory phenotype [136] Up-regulation of MHC I/II on tumour cells via gammaherpesvirus and endogenous retrovirus (ERV) 3–1 genes [150] Enhanced response to immune checkpoint via the induction of type III interferon response and the up-regulation of β2M and MHC I [170]	DC maturation via p73-mediated induction of DR5 [109] CD8⁺ T cell recruitment and activation through the CCR5 and CXCR3 increase triggered by mTOR-regulated metabolic activity [55] CD8⁺ T cell recruitment and activation via the STING pathway [129] NFAT-dependent CD8⁺ T cell recruitment and activation [134] CD8⁺ T and NK cell recruitment and activation via the cGAS/STING signalling [38] Mxd4/Myc-dependent induction of a T cell memory phenotype [136] SASP-dependent activation and infiltration of NK cells [33] Up-regulation of MHC I/II on tumour cells via gammaherpesvirus and endogenous retrovirus (ERV) 3–1 genes [150] PD-L1 up-regulation in tumour cells following SPOP degradation [163] Reversal of the immune-resistant tumour phenotype by blocking the SCP3-NANOG axis [154]	DC maturation via p73-mediated induction of DR5 [109] Up-regulation of MHC I/II on tumour cells via gammaherpesvirus and endogenous retrovirus (ERV) 3–1 genes [150]
**Metabolic reprogramming**	Increased glycolytic and oxidative phosphorylation activity, mitochondrial expansion, high ATP and ROS production in pancreatic ductal adenocarcinoma [17] Inhibition of mitochondrial apoptosis via MERC remodeling in TNBC cells [90] Reduced cholesterol biosynthesis via FOXM1 attenuation, toward inhibition of TNBC proliferation *in vitro* and *in vivo*[95]	MYC-dependent enhanced glucose, glutamine and amino acid metabolism [88] FBP1 stabilization-dependent decrease of glucose metabolism in pancreatic cancer cells [86] Increased glucose metabolism in CDK4/6 inhibitor-resistant breast cancer cells; reduced glucose metabolism in CDK4/6 inhibitor-sensitive cells upon treatment [87] Glucose intolerance, reduced insulin secretion and morphological alterations in pancreatic β-cells in young rats [94] Increased glycolytic and oxidative phosphorylation activity, mitochondrial expansion, high ATP and ROS production in pancreatic ductal adenocarcinoma [17] p53-dependent increased mitochondrial metabolism in melanoma cells [91] Reduced cholesterol biosynthesis via FOXM1 attenuation, toward inhibition of TNBC proliferation *in vitro *and *in vivo*[95]	Increased glycolytic and oxidative phosphorylation activity, mitochondrial expansion, high ATP and ROS production in pancreatic ductal adenocarcinoma [17]
**Adipocytes**	Fat mass reduction by inhibiting pRb phosphorylation in the mediobasal hypothalamus and enhancing lipid oxidation [182]	Inhibition of CAAT-induced breast tumour cell proliferation [183]	
**CAFs**	Reactivation of ERα signalling counteracting CAF‐mediated inhibition in breast cancer cells [22] CD63⁺ CAFs reduce efficacy in breast cancer cells via exosomal miR-20–mediated Rb downregulation [25]	Reactivation of ERα signalling counteracting CAF‐mediated inhibition in breast cancer cells [22] GPER-dependent increase of pro-inflammatory genes in breast CAFs [23] CAF enrichment in metastatic breast tumours [24] Reduced efficacy in TNBC cells co-cultured with fibroblasts [26]	

A major clinical challenge in the long-term management of ER-positive/HER2-negative advanced breast cancer is the development of CDK4/6 inhibitor resistance, which significantly limits the benefits of these drugs. In this context, novel CDK4/6 inhibitors have been evaluated as potential therapeutic strategies in combination with other treatments [207]. For instance, the phase 3 study DAWNA-2 reported the effectiveness of a selective CDK4/6 inhibitor, named dalpiciclib, used in combination with aromatase inhibitors in patients with ER-positive/HER2-negative advanced breast cancer [212, 213]. These results support evidence collected by the phase 3 study DAWNA-I (NCT03927456), thus encouraging more exploratory studies for the prediction of the the efficacy of dalpiciclib in combination with endocrine therapy (NCT04842617), even in patients displaying the failure of CDK4/6 inhibitors (NCT05861830). Several clinical trials are currently evaluating the efficacy of the multidrugs dalpiciclib-based regimens in certain subsets of patients, such as those with PIK3CA-mutated (NCT07207070) or SNF3 and SNF4-subtypes advanced breast cancers (NCT06612814, NCT06447623). Additionally, the phase 3 trial LEONARDA-1 has revealed that patients with ER-positive/HER2-negative locally advanced or metastatic breast cancer who experienced relapse or progression on prior endocrine therapy exhibited a prolonged survival upon treatment with the novel selective CDK4/6 inhibitor lerociclib [210]. With respect to metastatic TNBC, this subgroup of patients notoriously experiences unsatisfactory results from CDK4/6 inhibitors used as monotherapy, thus supporting further investigations aimed at elucidating key oncogenic drivers in this aggressive breast cancer subtype [211]. Consistent with these observations and evidence showing that TNBC is characterized by a high percentage of tumour-infiltrating lymphocytes, as well as high PD-L1 expression [212], a phase 2 trial revealed an enhancement of the anti-tumour immunity usingthe CDK4/6 inhibitor trilaciclib before chemotherapy in patients with metastatic TNBC (NCT02978716). Of note, trilaciclib received its first FDA approval in 2021 in order to protect patients with advanced-stage small cell lung cancer from chemotherapy-induced myelosuppression [213]. Considering that abemaciclib, palbociclib and ribociclib exert myelosuppressive effects, trilaciclib has also been evaluated as a potential strategy for treating breast cancer [214]. However, the phase 3 study PRESERVE-2 failed to demonstrate an improvement in the overall survival of metastatic TNBC patients treated with trilaciclib before gemcitabine and carboplatin (NCT04799249). In addition, several ongoing clinical studies are assessing the benefits of trilaciclib in combination with chemotherapy and ICIs in patients with ER-negative breast cancer (NCT06955156, NCT05978648 and NCT05862610).

In addition to the extensively tested combination of CDK4/6 inhibitors with ET regimens, an emerging therapeutic strategy consists of co-targeting the PI3K/AKT/mTOR pathway, which is reported to be highly activated in patients with ER-positive/HER2-negative advanced breast cancer [215]. In this context, ongoing clinical studies are evaluating the benefits of the coadministration of PI3K/AKT/mTOR inhibitors in patients with ER-positive/HER2-negative breast cancer following progression upon treatment with CDK4/6 inhibitors (NCT05392608, NCT05038735, NCT04650581 and NCT05501886). Additionally, based on the preclinical evidence supporting the use of the androgen receptor (AR) as a potential therapeutic approach for overcoming the resistance to standard-of-care ER and CDK4/6 inhibitors [216], a recent clinical trial evaluated the effectiveness of the selective androgen receptor modulator (SARM) enobosarm in combination with abemaciclib in patients with ER-positive/HER2-negative breast cancer who progressed upon CDK4/6 inhibitors (NCT05065411).

Besides breast cancers, the potential benefit of CDK4/6 inhibitor-based therapies has been evaluated in other types of tumours (NCT04902885, NCT03891784, NCT06552858). For instance, the efficacy of palbociclib in combination with the MEK inhibitor binimetinib is currently under evaluation for the management of advanced KRAS mutant non-small cell lung cancer and BRAF-mutant metastatic melanoma patients (NCT03170206, NCT04720768). CDKs, in particular the CDK6 isoform, are key-regulators of hematopoietic processes, thus explaining the dose-limiting hematologic toxicity frequently observed in patients treated with CDK4/6 inhibitors [221–223]. In this context, the efficacy and safety of novel CDK4-selective inhibitors, such as atirmociclib, are currently under evaluation in the phase 3 “FourLight-3” study in ER-positive/HER2-negative advanced breast cancer (NCT06760637) [220]. A detailed summary of the most relevant and recent clinical studies, highlighting the potential therapeutic usefulness of CDK4/6 inhibitors, is provided in Table2.

A further critical challenge remains the identification of molecular biomarkers capable of predicting the therapeutic response to CDK4/6 inhibitors in defined patient subgroups. In this context, the analysis of fresh biopsies collected from several breast cancer patients undergoing CDK4/6-inhibitor regimens has revealed poor responses associated with genetic alterations including genes such as PTEN, AKT1, RAS, CYCLIN-E1 and FAT-1 [225–229]. Additionally, liquid biopsy has emerged as a promising tool for the discovery of novel molecular biomarkers. In particular, advanced breast cancer patients resistant to CDK4/6-inhibitors exhibit elevated circulating levels of certain miRNAs such as miR-21, miR-200a and miR-200b [226]. Other nucleic acids, like circulating tumour DNAs and long non-coding RNAs, are currently under evaluation as promising biomarkers of prognosis and response to treatments (NCT05826964, NCT06307249). Novel insights are expected from ongoing clinical trials such as the Roswell Park Ciclib Study, which analyzes fresh body fluids and biopsy samples from approximately 700 patients with advanced or metastatic breast cancer treated with CDK4/6 inhibitors (NCT04526587, NCT05977036, NCT05826964, NCT03310879). Recent studies are exploring the microbiome as a potential key regulator of the actions of CDK4/6 inhibitors. In this vein, the Ciclibiome observational study aims to identify gut microbial, immune and metabolic biomarkers associated with the response to the CDK4/6 inhibition in breast cancer patients (NCT06171360).

Based on preclinical evidence showing that CDK4/6 inhibitors can also induce several effects other than cell cycle arrest, emerging data suggest that CDK4/6 inhibitors may exert unintended pro-tumourigenic effects in stromal components of the TME, particularly in CAFs. In addition, CDK4/6 inhibition has been shown to induce the SASP, characterized by the release of proinflammatory cytokines, growth factors and proteolytic enzymes, which can promote a TME conducive to the progression of tumours and the failure of therapeutics. Importantly, these stimulatory effects triggered by CDK4/6 inhibitors introduce new layers of complexity, particularly in predicting the response to treatments and in managing adverse effects within the TME. In addition, corroborating the role of the TME in determining the response to CDK4/6 inhibition, recent transcriptomic and immunophenotyping studies have identified distinct TME signatures characterized by specific immune cell subsets and inflammatory profiles in breast cancer patients treated with CDK4/6 inhibitors. These immune-related profiles have been linked to differential responses to therapeutic agents and thus could be considered as valuable predictive biomarkers. These insights underscore the importance of incorporating TME features into therapeutic decision-making, providing also a compelling rationale for the development of tailored combination strategies, particularly those that integrate CDK4/6 inhibitors with immunotherapeutic agents or TME-targeting modulators.

Overall, a deeper understanding of the mechanistic actions of CDK4/6 inhibitors will be essential for designing more effective and personalized treatments. This includes their rational combination with ICIs, senomorphics, or CAF-targeting approaches, such as engineered nanoparticles capable of sequestering CAF-derived exosomal miR-20, recently developed by Sun et al. [25]. Such strategies may extend beyond the current indications for ER-positive/HER2-negative breast cancer, broadening the clinical utility of CDK4/6 inhibitors across diverse tumour subtypes and therapeutic contexts. Moreover, improved biological insights could enhance patient selection and help overcome resistance mechanisms, which remain major barriers to durable clinical benefit.

**Table 2:** Clinical trials investigating emerging therapeutic strategies targeting CDK4/6

Trial	CDK4/6 inhibitor	Study design	Disease	Survival outcomes	Status
DAWNA-1 (NCT03927456)	Dalpiciclib	Phase 3 randomized, quadruple-blind, placebo-controlled; dalpiciclib/placebo + fulvestrant	ER +/HER2-advanced breast cancer with disease progression during or after previous endocrine therapy	PFS, OS, ORR, DoR, CBR	Closed
NCT06133088	Dalpiciclib	Phase 2; dalpiciclib + fluvestrant + compound gossypol acetate tablets	ER +/HER2-breast cancer after CDK4/6 treatment failed	PFS, CBR, DoR, TTR, OS	Recruiting
DAWNA-2 (NCT03966898)	Dalpiciclib	Phase 3 randomized, double-blind, placebo-controlled; dalpiciclib + letrozole or anastrozole *vs* placebo + letrozole or anastrozole as first-line treatment	ER +/HER2-advanced breast cancer	PFS, OS, ORR, DoR, CBR	Closed
NCT04842617	Dalpiciclib	Phase 3 randomized, quadruple-blind, placebo-controlled; dalpiciclib + letrozole or anastrozole *vs* placebo + letrozole or anastrozole	ER +/HER2-advanced breast cancer	IDFS, DFS, DDFS	Active, not recruiting
DAWNA-FES (NCT05861830)	Dalpiciclib	Phase 3 randomized; dalpiciclib with endocrine therapy vs dalpiciclib with chemotherapy	ER +/HER2-advanced breast cancer after CDK4/6 Inhibitor treatment failure using 18F-FES PET/CT	PFS, OS, ORR, DCR	Recruiting
NCT07207070	Dalpiciclib	Phase 3 randomized; JS105 + dalpiciclib and fulvestrant vs dalpiciclib and fulvestrant	PIK3CA-mutated ER&/HER2-recurrent or metastatic breast cancer	PFS, OS, ORR, DCR	Not yet recruiting
NCT06612814	Dalpiciclib	Phase 3 randomized; dalpiciclib + fluzoparib and endocrine therapy vs dalpiciclib + endocrine therapy	SNF3 subtype ER +/HER2-recurrent or metastatic breast cancer	PFS, OS, ORR, DCR	Active, not recruiting
NCT06447623	Dalpiciclib	Phase 3 randomized; apatinib + dalpiciclib and endocrine therapy vs dalpiciclib + endocrine therapy	SNF4 subtype ER +/HER2-recurrent or metastatic breast cancer	PFS, OS, ORR, CBR	Recruiting
NCT04486911	Dalpiciclib	Phase 2; pyrotinib maleate + dalpiciclib + letrozole	Stage II–III TNBC	BORR, RCB, OS, DFS	Active, not recruiting
NCT06556862	Dalpiciclib	Phase 2; dalpiciclib + HDACi + endocrine therapy	HR +/HER2-advanced breast cancer	PFS, ORR, CBR, DCR, DoR, OS	Not yet recruiting
NCT05586841	Dalpiciclib	Phase 1; Dalpiciclib + chidamide	HR +/HER2-advanced breast cancer after failure of CDK4/6 inhibitor:	ORR, PFS	Recruiting
NCT06225921	Dalpiciclib	Phase 1; dalpiciclib + adebrelimab	Resectable esophageal squamous cell carcinoma	ORR, PFS, OS	Recruiting
NCT06109207	Dalpiciclib	Phase 1; camrelizumab + dalpiciclib	Resectable head and neck squamous cell carcinomas	PFS, ORR, OS	Recruiting
LEONARDA-1 (NCT05054751)	Lerociclib	Phase 3 randomized, quadruple-blind, placebo-controlled; lerociclib/placebo + fulvestrant	ER +/HER2-locally advanced or metastatic breast cancer	PFS, OS, ORR, CBR, DOR, DCR	Completed
NCT02978716	Trilaciclib	Phase 3 randomized; gemcitabine/carboplatin vs trilaciclib + gemcitabine/carboplatin (days 1 and 8) vs trilaciclib (days 1, 2, 8 and 9) + gemcitabine/carboplatin (days 2 and 9)	Metastatic TNBC	PFS, OS, DOR, BOR	Terminated
PRESERVE-2 (NCT04799249)	Trilaciclib	Phase 3 randomized, double-blind, placebo-controlled; trilaciclib/placebo + gemcitabine and carboplatin	Metastatic TNBC previously treated or not with a PD-1/PD-L1 inhibitor	PFS, OS	Completed
NCT06955156	Trilaciclib	Phase 2; trilaciclib + anti-PD-1 antibody + chemotherapy	Locally advanced TNBC	EFS, pCR	Recruiting
NCT05978648	Trilaciclib	Phase 2; trilaciclib + chemotherapy	TNBC and ER-/PR-/Her2 + breast cancer	-	Recruiting
NCT05862610	Trilaciclib	Phase 2 randomized; trilaciclib plus chemotherapy	TNBC	-	Not yet recruiting
NCT04902885	Trilaciclib	Phase 3 randomized, quadruple-blind, placebo-controlled; placebo vs triaciclib	Extensive-stage small cell lung cancer	ORR, DCR	Completed
PRESERVE-1 (NCT04607668)	Trilaciclib	Phase 3 randomized, double-blind, placebo-controlled; triaciclib/placebo + FOLFOXIRI/bevacizumab	Metastatic colorectal cancer	ORR, OS, PFS	Terminated
NCT06151262	Trilaciclib	Phase 2; trilaciclib + mFOLFIRINOX	Advanced pancreatic cancer	PFS, OS	Recruiting
JUINIPER (NCT02152631)	Abemaciclib	Phase 3 randomized; abemaciclib vs erlotinib	Stage IV NSCLC with a detectable KRAS mutation who have progressed after platinum-based chemotherapy	ORR, PFS	Active, not recruiting
CYCLONE 3 (NCT05288166)	Abemaciclib	Phase 3 randomized; double-blind, placebo-controlled; abemaciclib/placebo + abiraterone acetate	Metastatic hormone-sensitive prostate cancer	rPFS	Active, not recruiting
VERU-024 (NCT05065411)	Abemaciclib	Phase 3 randomized; enobosarm + abemaciclib vs non-steroidal or steroidal AI or fulvestrant	ER +/HER2-MBC in patients who have shown previous disease progression on an estrogen blocking agent plus palbociclib	PFS, OR	Terminated
ELAINEIII (NCT05696626)	Abemaciclib	Phase 3 randomized; abemaciclib + lasofoxifene/fulvestrant	Locally advanced or metastatic ER +/HER2-breast cancer with ESR1 mutation	PFS, OS, ORR, CBR, DOR	Recruiting
CYCLONE 2 (NCT03706365)	Abemaciclib	Phase 2/3 randomized, double-blind, placebo-controlled; abemaciclib + abiraterone acetate and prednisone	Metastatic castration-resistant prostate Cancer	rPFS, OS, DOR, ORR	Active, not recruiting
CAMBRIA-2 (NCT05952557)	Abemaciclib	Phase 3 randomized; abemaciclib + endocrine therapy vs abemaciclib + camizestrant	ER +/HER2-early breast cancer	IDFS, DRFS, OS	Recruiting
lidERA Breast Cancer (NCT04961996)	Abemaciclib	Phase 3 randomized; abemaciclib + giredestrant, then giredestrant vs giredestrant	ER +/HER2-early breast cancer	IDFS, OS, DRFI, DFS, LRRFI	Active, not recruiting
NCT05891093	Abemaciclib	Phase 3 randomized; abemaciclib + fluzoparib	ER +/HER2-SNF3-subtype early breast cancer	DRFS, OS	Recruiting
NCT03310879	Abemaciclib	Phase 2; abemaciclib in different genetic subsets of patients	Solid tumours harboring genetic alterations in genes encoding D-type cyclins or amplification of CDK4 or CDK6	PFR, ORR	Recruiting
NCT05617885	Abemaciclib	Phase 2; abemaciclib + darolutamide	High-risk prostate cancer	ORR, PFS	Active, not recruiting
NCT05372640	Abemaciclib	Phase 1; abemaciclib + ZEN003694	NUT carcinoma, breast cancer and other solid tumours	DOR, PFS, ORR, CBR	Recruiting
NCT03675893	Abemaciclib	Phase 2; abemaciclib + Letrozole +/-metformin or zotatifin or gedatolisib	Endometrial or low-grade serous ovarian cancer	PFS, OS	Recruiting
NCT05774899	Abemaciclib	Phase 2; CB-103 + lenvatinib or abemaciclib	NOTCH activated adenoid cystic carcinoma	DOR, PFS, ORR, OS	Recruiting
NCT04750928	Abemaciclib	Phase 2	Neurofibromatosis type I (NF1) related atypical neurofibromas	Stable disease rate	Recruiting
CAPItello-292 (NCT04862663)	Abemaciclib or palbociclib or ribociclib	Phase 2b/3 randomized; fulvestrant + investigator’s choice of CDK4/6 inhibitor vs capivasertib + fulvestrant + investigator’s choice of CDK4/6 inhibitor	ER +/EGFR2-locally advanced, unresectable or metastatic breast cancer	DOR, PFS, ORR, OS, CBR	Recruiting
SERENA-6 (NCT04964934)	Abemaciclib or palbociclib or ribociclib	Phase 3, double-blind, randomized; AZD9833 + CDK4/6 inhibitor vs anastrozole or letrozole + CDK4/6 inhibitor	HR +/HER2-metastatic breast cancer	PFS, OS, ORR	Active, not recruiting
pionERA Breast Cancer (NCT06065748)	Abemaciclib or palbociclib or ribociclib	Phase 3 randomized; giredestrant + investigator’s choice of CDK4/6 inhibitor	HR +/HER2-metastatic breast cancer resistant to adjuvant endocrine therapy	CBR, ORR, OS	Recruiting
EvoPAR-BR01 (NCT06380751)	Abemaciclib or palbociclib or ribociclib	Phase 3 randomized; CDK4/6 inhibitor + endocrine therapy vs CDK4/6 inhibitor + camizestrant	ER +/HER2-; BRCA1, BRCA2 or PALB2 mutation advanced breast cancer	PFS, OS, ORR	Recruiting
NCT06062498	Abemaciclib or palbociclib or ribociclib	Phase 2; elacestrant + CDK4/6 inhibitor	ER +/HER2-advanced and metastatic breast cancer	DOR, OS, PFS	Recruiting
VIKTORIA-2 (NCT06757634)	Palbociclib or ribociclib	Phase 3 randomized; gedatolisib + fulvestrant + palbociclib or ribociclib	ER +/HER2-advanced and metastatic breast cancer	OS, DOR, ORR, CBR	Recruiting
NCT05216432	Palbociclib or ribociclib	Phase 1; RLY2608 + fulvestrant + palbociclib or ribociclib	Advanced solid tumours or advanced breast cancer	DCR, DOR, ORR, CBR	Recruiting
VERITAC-3 (NCT05909397)	Palbociclib	Phase 3 randomized; palbociclib + ARV-471 vs palbociclib + letrozole	ER +/HER2-advanced and metastatic breast cancer	PFS, ORR, OS, CBR	Active, not recruiting
NCT04920708	Palbociclib	Phase 2 randomized; palbociclib + fulvestrant ± ipatasertib	Metastatic ER +/HER2-breast cancer patients without ctDNA suppression	PFS	Recruiting
VIKTORIA-1 (NCT05501886)	Palbociclib	Phase 3 randomized; gedatolisib + palbociclib + fulvestrant vs gedatolisib + fulvestrant	ER +/HER2-advanced and metastatic breast cancer with or without PIK3CA mutations	PFS, OS, ORR, DOR, TTR, CBR	Recruiting
INAVO120 (NCT04191499)	Palbociclib	Phase 3 randomized, double-blind, placebo-controlled; inavolisib/placebo + palbociclib + fulvestrant	ER +/HER2-advanced and metastatic breast cancer with PIK3CA Mutations	PFS, OS, ORR, DOR, BOR, CBR	Active, not recruiting
AMEERA-5 (NCT04478266)	Palbociclib	Phase 3 randomized, double-blind; letrozole + palbociclib vs amcenestrant + palbociclib	ER +/HER2-advanced and metastatic breast cancer with PIK3CA mutations	PFS, OS, DOR	Terminated
PIKALO-2 (NCT07174336)	Palbociclib	Phase 3 randomized, double-blind, placebo-controlled; LY4064809/placebo + palbociclib + endocrine therapy	ER +/HER2-advanced and metastatic breast cancer with PIK3CA mutations	PFS, OS, DOR, ORR, TTR, DCR	Not yet recruiting
NCT07061717	Palbociclib	Phase 3 randomized; palbociclib + endocrine therapy + hydroxychloroquine	ER +/HER2-advanced/metastatic breast cancer	PFS, OS, ORR, CBR	Not yet recruiting
SERENA-4 (NCT04711252)	Palbociclib	Phase 3 randomized, double-blind; AZD9833 + palbociclib vs anastrozole + palbociclib	ER +/HER2-breast cancer	PFS, OS, ORR, DOR	Active, not recruiting
INAVO123 (NCT06790693)	Palbociclib	Phase 3 randomized, double-blind, placebo-controlled; inavolisib/placebo + letrozole + palbociclib	ER +/HER2-advanced breast cancer	PFS, OS, CBR, DOR, ORR	Recruiting
NCT03170206	Palbociclib	Phase 1; palbociclib + binimetinib	Advanced KRAS Mutant NSCLC	PFS	Recruiting
NCT03065062	Palbociclib	Phase 1; palbociclib + gedatolisib	Advanced squamous cell lung, pancreatic, head and neck and other solid tumours	PFS, ORR	Recruiting
CELEBRATE (NCT04720768)	Palbociclib	Phase 2; palbociclib + encorafenib + binimetinib	BRAF-mutant metastatic melanoma	PFS, OS	Recruiting
NCT05266105	Palbociclib	Phase 1; palazestrant in combination with palbociclib	Advanced or metastatic ER +/HER2-breast cancer	-	Active, not recruiting
NCT05935748	Palbociclib	Phase 2; NKT2152 + palbociclib/palbociclib + sasanlimab	Advanced or metastatic clear cell renal cell carcinoma	PFS, DOR, OS, CBR,	Active, not recruiting
NCT06126276	Palbociclib	Phase 2; palbociclib + neratinib	HER2 + solid tumours	PFS, OS	recruiting
NCT04841148	Palbociclib	Phase 2; avelumab or hydroxychloroquine ± palbociclib	Dormant breast cancer	RFS	recruiting
NCT05865132	Palbociclib	Phase 2; palbociclib + afatinib	Advanced squamous carcinoma of esophagus or gastroesophageal junction	ORR, DCR, OS, PFS	recruiting
NCT03959891	Palbociclib	Phase 1; fulvestrant + ipatasertib + palbociclib	ER +/HER2-advanced breast cancer	PFS, ORR, OS	Active, not recruiting
NCT03024489	Palbociclib	Phase 2; palbociclib + cetuximab + intensity modulated radiation therapy (IMRT)	Locally advanced squamous cell carcinoma	ORR	Active, not recruiting
NCT06947811	Palbociclib	Phase 2; almonertinib + palbociclib	Advanced solid tumours harboring KRAS mutations	ORR, PFS	Recruiting
NCT03709680	Palbociclib	Phase 2; palbociclib + irinotecan + temozolomide	Pediatric refractory solid tumours	PFS, OS, DoR, EFS	Active, not recruiting
OPERA-2 (NCT07085767)	Ribociclib	Phase 3 randomized, double-blind; palazestrant + ribociclib + placebo vs letrozole + ribociclib + placebo	ER +/HER2-advanced breast cancer	PFS, OS, DOR, ORR, CBR	Not yet recruiting
KontRASt-03 (NCT05358249)	Ribociclib	Phase 2; JDQ443 + ribociclib	Advanced solid tumours harboring the KRAS G12C mutation	ORR, DCR, DoR, PFS, OS	Active, not recruiting
NCT03114527	Ribociclib	Phase 2; everolimus + ribociclib	Advanced dedifferentiated liposarcoma (DDL) and leiomyosarcoma (LMS)	ORR, PFS, OS	Active, not recruiting
NCT05870579	Ribociclib	Phase 1; [177Lu]Lu-NeoB + ribociclib + fulvestrant	ER +/HER2- and GRPR + advanced breast cancer	ORR, CBR, DoR, OS	Recruiting
NCT03090165	Ribociclib	Phase 2; ribociclib + bicalutamide	Advanced AR + TNBC	CBR, ORR, PFS, OS	Recruiting
NCT05508906	Ribociclib or atirmociclib	Phase 1; palazestrant + ribociclib or atirmociclib	ER +/HER2-breast cancer	CBR, DoR	Recruiting
FourLight-3 (NCT06760637)	Atirmociclib (selective CDK4 inhibitor)	Phase 3 randomized; atirmociclib + letrozole vs investigator’s choice of CDK4/6 inhibitor + letrozole	ER +, HER2-advanced/metastatic breast cancer	PFS, OS, ORR	Recruiting

## Data Availability

Not applicable. No datasets were generated or analysed during the current study.

## Abbreviations

AMBRA1: Activating molecule in beclin1-regulated autophagy protein 1
AMPK: AMP-activated protein kinase
AMPKα: AMP-activated protein kinase catalytic subunit alpha
ANGPTL4: Angiopoietin-like 4
APCs: Antigen-presenting cells
AR: Androgen receptor
ATG5: Autophagy related 5
ATP: Adenosine triphosphate
ATP6AP2: ATPase H + transporting accessory protein 2
ATR: Ataxia telangiectasia and Rad3-related protein
BOR: Best overall response
BORR: Best overall response rate
BRAF: V-Raf murine sarcoma viral oncogene homologue B
CAAT: Cancer-associated adipose tissue
CAFs: Cancer-associated fibroblasts
CBR: Clinical benefit rate
CCNA2: Cyclin A2
CCNB1: Cyclin B1
CCNB2: Cyclin B2
CCND3: Cyclin D3
CCNE2: Cyclin E2
CDK2: Cyclin-dependent kinase 2
CDK4/6: Cyclin-dependent kinases 4 and 6
CDKN2B: Cyclin-dependent kinase inhibitor 2B
CDKs: Cyclin-dependent kinases
cGAS–STING pathway: Cyclic GMP-AMP synthase–stimulator of interferon genes
CHK1: Checkpoint kinase 1
COX-2: Cyclooxygenase-2
CSF2: Colony-stimulating factor 2
ctDNA: Circulating tumour DNA
DCR: Disease control rate
DCR2: Decoy receptor 2
DCs: Dendritic cells
DDFS: Distant disease-free survival
DEC1: Differentiated embryo-chondrocyte expressed gene 1
DFS: Disease-free survival
DoR: Duration of response
DR5: Death receptor 5
DRFI: Distant recurrence-free interval
DRFS: Distant relapse-free survival
EBV: Epstein–Barr virus
ECM: Extracellular matrix
EFS: Event-free survival
EGFR2: Human epidermal growth factor receptor 2
EMA: European Medicines Agency
ER: Oestrogen receptor
ESR1: Estrogen receptor 1
FDA: Food and Drug Administration
FOXM1: Forkhead Box M1
GPER: G protein-coupled oestrogen receptor
GRPR: Gastrin releasing peptide receptor
HER2: Human epidermal growth factor receptor 2
HK2: Hexokinase 2
ICIs: Immune checkpoint inhibitors
IDFS: Invasive disease-free survival
IFN-γ: Interferon gamma
IGFBP2: Insulin-like growth factor-binding protein 2
IL-15: Interleukin-15
IL-17A: Interleukin-17A
IL-2: Interleukin-2
KRAS: Kirsten Rat Sarcoma Virus
KSHV: Kaposi’s sarcoma-associated herpesvirus
LC3: Microtubule-associated protein 1 light chain 3
LRRFI: Loco-regional recurrence-free interval
MAPK: Mitogen-activated protein kinase
MBC: Male breast cancer
MDSCs: Myeloid-derived suppressor cells
MEK: Mitogen-activated protein kinase kinase
MERCs: Mitochondria–endoplasmic reticulum contacts
MHC: Major histocompatibility complex
MIF: Macrophage migration inhibitory factor
miR-33a: MicroRNA-33a
mTOR: Mechanistic target of rapamycin
MXD4: MAX dimerization protein 4
NFAT: Nuclear factor of activated T cells
NK: Natural killer (cell)
NSCLC: Non-small cell lung cancer
NUT: Nuclear protein in testis
ORR: Overall response rate
OS: Overall survival
PALB2m: Partner and localizer of BRCA2 mutation
pCR: Pathological complete response
PD-L1: Programmed death-ligand 1
PFS: Progression-free survival
PGRN: Progranulin
PI3K: Phosphatidylinositol 3-kinase
PIK3CA: Phosphatidylinositol-4,5-bisphosphate 3-kinase, catalytic alpha
PKA: Protein kinase A
PP5: Protein phosphatase 5
PPAR-γ: Peroxisome proliferator-activated receptor gamma
Rb: Retinoblastoma protein
RFS: Recurrence-free survival
ROS: Reactive oxygen species
rPFS: Radiographic progression-free survival
SASP: Senescence-associated secretory phenotype
SCP3-NANOG: Synaptonemal complex protein 3-homeobox protein NANOG
SNF3: Serine/arginine-rich splicing factor 3
SPOP: Speckle-type POZ protein
STAT3: Signal transducer and activator of transcription 3
STAT5A: Signal transducer and activator of transcription 5A
STING: Stimulator of interferon genes
TAMs: Tumour-associated macrophages
TANs: Tumour-associated neutrophils
TFEB/TFE3: Transcription factor EB/transcription factor E3
TGF-β: Transforming growth factor beta
TIME: Tumour immune microenvironment
TME: Tumour microenvironment
TNBC: Triple-negative breast cancer
Tregs: Regulatory T cells
TTR: Time in therapeutic range
UCP1: Uncoupling protein 1
WAT: White adipose tissue
β2M: Human β2-microglobu
